# Usage Patterns of Telepsychology and Face-to-Face Psychotherapy: Clients’ Profiles and Perceptions

**DOI:** 10.3389/fpsyg.2022.821671

**Published:** 2022-07-06

**Authors:** Beatriz Sora, Rubén Nieto, Adrian Montesano, Manuel Armayones

**Affiliations:** ^1^Department of Psychology, Faculty of Education Sciences and Psychology, University of Rovira i Virgili, Tarragona, Spain; ^2^Faculty of Psychology and Education Sciences, eHealth Center, Open University of Catalonia, Barcelona, Spain

**Keywords:** telepsychology, patients’ profiles, telepsychology advantages, telepsychology barriers, telepsychology efficacy

## Abstract

**Background:**

Currently, most people who might need mental health care services do not receive them due to a number of reasons. Many of these reasons can be overcome by telepsychology, in other words, the use of ICT technologies for therapy (e.g., phone, videoconferencing, and apps); given that it facilitates access to specialized interventions. In fact, telepsychology is currently offered as an active service in many psychotherapy centers. However, its usage, how it is perceived, and who uses it are still largely unknown.

**Objective:**

The aim of this study was (1) to determine if any pattern exists in the usage of telepsychology and face-to-face psychology, (2) to clarify people’s perception of telepsychology in terms of the advantages, barriers and efficacy of online psychotherapy, and (3) to examine usage patterns in terms of individual characteristics and identify patients’ profiles.

**Methods:**

An online survey was conducted on a convenience sample of 514 subjects recluted by using an online advertisement. The inclusion criteria were: (1) to be older than 18 years old and (2) to answer completely the questionnaire. Cluster analysis, ANOVAs, and discriminant analysis were performed to test our research objectives.

**Results:**

Three usage clusters were found: (1) face-to-face psychotherapy (57%; *n* = 292); (2) non-therapy (36.8%; *n* = 189); and (3) combined face-to-face psychotherapy and telepsychology (6.4%; *n* = 33). In addition, the perception of telepsychology varied among usage clusters, but a common perception emerged about the main telepsychology advantages, barriers and efficacy. Finally, the results showed that personal characteristics differentiated people in each of these clusters.

**Conclusion:**

The most common form of access to psychotherapy is the face-to-face form but the second way of delivery was a combination between face to face and online psychotherapy (research objective 1). People who combine face to face with online psychotherapy perceives this last as more efficient and with less barriers to access (research objective 2). Finally, some characteristic as eHealth experience and sociodemographic variables can help to identify people that will attend telepsychology initiatives (research objective 3). These clusters provide insight into opportunities for face-to-face and online patient engagement strategies.

## Introduction

Every year a high proportion of the population needs access to mental health services. For example, the World Health Organization (2001) stated that one in every four people develops one or more mental or behavioral disorders at some stage in life. Despite these numbers, not all people have adequate access to intervention. Figures vary widely depending upon the study and definition used. As an example, Alonso et al. (2007) found in a representative sample of the European population that 6.5% of people had required mental health care (i.e., a 12-month disorder that was disabling or had led to the use of health services in the previous year), and it was estimated that 3.1% of the adult population had an unmet need for mental health care.

Unfortunately, most people do not receive the specialized mental health care they require (Wang et al., 2007). Reasons for not receiving this care are varied, some of the most important of which are: stigma, socio-cultural factors, type of diagnosis, geographical distances, financial cost, lack of knowledge regarding mental health problems or available services, and certain beliefs (Simpson et al., 2009; Tirintica et al., 2018). However, information and communication technologies (ICT) have a great potential to overcome these barriers and facilitate access to interventions, specially telepsychology, which has emerged in recent years as an alternative to traditional face-to-face interventions. This has been defined by the American Psychological Association (APA) as “the provision of psychological services using telecommunication technologies.” Telepsychology includes the use of different electronic tools, ranging from telephones and fiber optics to interactive satellite video, in order to deliver healthcare (Rees and Haythornthwaite, 2004). This paper focuses specifically on videoconferencing technology, which enables people to see and talk to each other as if they were in the same room despite actually being apart. In this respect, there is a large amount of research literature supporting its efficacy to treat certain mental health problems and its comparability to face-to-face provided mental health care (Reese et al., 2015; Varker et al., 2019). When conducted properly, telepsychology seems to be as effective as traditional psychotherapy for clients with mood disorders, anxiety, substance abuse disorders, for young children and even for people with dementia or psychotic patients (Turvey, 2018; Berryhill et al., 2019). Therefore, videoconferencing psychotherapy has the potential to improve access to and quality of specialized care for a broad range of underserved clients. The full specification of optimal functioning, however, has yet to be determined considering routine practice settings and clients’ perspective.

Given the advantages associated with telepsychology, many public and private psychotherapy clinics offer such services in their routine practice around the world. For instance, in Spain, González-Peña et al. (2017) pointed out that 26.66% of psychologists were actually offering psychotherapy through videoconferencing, and that 60.49% reported to be in favor of offering this in the near future. However, the other side of the coin has been overlooked in the literature. Little is known about how this form of therapy delivery (telepsychology) is perceived and how it is used by patients compared to traditional face-to-face psychotherapy. Indeed, research on telepsychology from the patient perspective is very limited and only a few studies can be found on this topic (e.g., Germain et al., 2010). As Eichenberg, Wolters and Brähler stated, “there is a broad range of modern media that can be applied in the provision of health information and the treatment of mental disorders. But even though these means have been found to be effective in the treatment of certain mental disorders, the usage and demand of web-based information and interventions offer is still largely unknown” (Eichenberg et al., 2013). From a broad perspective of eHealth, the literature states that eHealth technologies can be socially perceived in two different ways. The first is characterized by enthusiasm and high hopes for new health technologies, and the other by a criticism of the development of these technologies, questioning their usefulness, highlighting the potential danger to confidentiality, and suggesting an impairment of the physician-patient relationship through the dehumanization of care (e.g., Williams et al., 2013; Jacomet et al., 2020). It seems plausible to apply this assumption to telepsychology from a theoretical perspective, but there is not yet any empirical evidence to support this. Thus, we have to conclude that there is no clear knowledge about people’s perception of telepsychology nor their usage of it.

Furthermore, and in parallel, research on conventional face-to-face psychology has pointed out that the usage of therapy depends on personal characteristics, showing differences in usage among patients (Seija et al., 2001; Lakey and Ondersma, 2008). In other words, a significant amount of research has studied the face-to-face therapy patient profile and its association with adherence to and the success of therapy (e.g., Delgadillo et al., 2016; Bernecker et al., 2017). Others have paid attention to the profile of people that attend face-to-face psychotherapy (e.g., Briffault et al., 2008), pointing out key factors such as sex, age, education level (Olfson and Pincus, 1994; Olfson et al., 2002), and ethnic (Chen and Rizzo, 2010) or marital status (Olfson et al., 2002). Regarding telepsychology, we are aware of only a few studies addressing clients’ perspectives. In a German sample, Eichenberg et al. (2013) evidenced that media-supported psychotherapy was not so well-accepted with mental disorders in need of treatment. Conventional psychotherapy is preferred by most Germans, even though the willingness to use technological adjuncts of therapy increased if the device was already used. The profile of patients who reported a willingness to use telepsychology was: men, young adults, singles, with a final degree and an income of over 2,500 euros per month. Finally, the results also showed significant relationships between the use of media-assisted therapy and the use of different sources to obtain health information (Williams et al., 2013). While it is true that literature about acceptability of internet delivery interventions for mental health has pointed out that, in general, clients hold a positive attitude, with moderate to high levels of satisfaction (Berry et al., 2016; Andersson, 2018; Bennett et al., 2020), it is also true that continue to be smaller than in face-to-face psychotherapy. Research indicated better acceptability among young women with higher educational level, and among those perceiving more barriers to access specialized psychological care (Moskalenko et al., 2020). Users prefer guided interventions or videoconference over unguided self-applied web-based packages (Martí-Noguera, 2022). Nevertheless, there are only a few studies discerning the relationship between usage patterns, acceptability, and efficacy of eHealth interventions, and with severe methodological frailties (small and heterogeneous samples, descriptive results, scarcity of psychometrics) and, therefore, clients’ perspective and preferences remains underrepresented in research. Further and unfortunately, there does not appear to be any study that has examined the profile of telepsychology patients in Spain. In fact, in Spain, there is a striking absence of quantitative data on the frequencies and determinants of access to psychotherapy.

In conclusion, telepsychology is currently offered among routine services in psychotherapy centers. But even though it has been found to be effective in the treatment of certain mental disorders (Reese et al., 2015; Varker et al., 2019), its usage, how it is perceived, and who uses it are still largely unknown. This knowledge is indispensable to identify the critical factors that allow to promote the use of telepsychology and its efficacy in a wider range of mental disorders. Hence, the present study aimed (1) to examine how telepsychology is used in relation to face-to-face psychotherapy and determine whether any usage pattern can be identified (2) to clarify people’s perception of telepsychology, in terms of benefits, barriers and efficacy, and (3) to provide information about the usage of tele- and conventional psychotherapy patterns in terms of individual characteristics (e.g., sex, age, education, and economic status) and eHealth skills (understood as the subjective perception that people have of their skills and knowledge on eHealth and previous eHealth experience).

## Materials and Methods

### Procedure and Sample

An online survey was conducted through the Qualtrics platform. This was disseminated by using an online advertisement published on our university’s website. The ad explained the research project, explained its main objective, and asked for volunteers who might be willing to participate in our research by taking an open online survey. In order to increase response rates, the researchers sent this link along with a brief summary of the research project to their contacts *via* email.

A total of 568 persons entered the system; of these, 54 did not complete the survey and were excluded. Table 1 presents the characteristics of the 514 participants in detail.

**TABLE 1:** Characteristics of participants in the study.

	Total			Cluster 1: face-to-face psychotherapy			Cluster 2: non-therapy			Cluster 3: combined therapy		
	Mean	SD	Freq. (N)	Mean	SD	Freq. (N)	Mean	SD	Freq. (N)	Mean	SD	Freq. (N)
Sex	–	–		–	–		–	–		–	–	
Man			20.2% (104)			19.5% (57)			22.2% (42)			15.2% (5)
Woman			79.8% (410)			80.5% (235)			77.8% (147)			84.8% (28)
Age	36.27	1.35		37.30	9.90		34.72	10.89		36.00	10.20	
Education	–	–		–	–		–	–		–	–	
No studies			0.4% (2)			0% (0)			0.5% (1)			3% (1)
Primary studies			2.7% (14)			2.1 (6)			4.2% (8)			0% (0)
Secondary studies			27% (139)			30.8% (90)			23.8% (45)			12.1% (4)
University studies			43.2% (222)			43.2% (126)			42.3% (80)			48.5% (16)
Post-graduate studies			26.7% (137)			24% (70)			29.1% (55)			36.4% (12)
Economic status	3.02	1.38										
Less than €600			17.1% (88)			17.5% (51)			16.4% (31)			18.2% (6)
€600–999			16.7% (86)			17.5% (51)			15.9% (30)			15.2% (5)
€1,000–1,499			26.7% (137)			29.5 (86)			23.8% (45)			18.2% (6)
€1,500–1,999			20.6% (106)			19.9% (58)			21.7% (41)			21.2% (7)
€2,000–3,000			10.7% (55)			9.9% (29)			11.6% (22)			12.1% (4)
More than €3,000			3.5% (18)			2.7% (8)			4.2% (8)			6.1% (2)
eHealth skills	3.31	1.01		3.27	1.01		3.28	0.96		3.73	1.17	
eHealth experience	0.54	0.50		0.85	0.85		0.88	0.78		2.42	1.17	
I have visited web pages			53.5% (275)			52.4% (153)			54% (102)			
I have communicated with professionals by email or chat			10.5% (54)			9.6% (28)			2.1% (4)			60.6% (20)
I have communicated with professionals through videoconferences			4.5% (23)			1% (3)			0% (0)			66.7% (22)
I have used some mobile application (apps)			8% (41)			6.8% (20)			7.9% (15)			60.6% (20)
I have contacted professionals by telephone (telephone therapy)			6.8% (35)			6.8% (20)			1.6% (3)			18.2% (6)
Others			12.3% (63)			8.9% (26)			19% (36)			36.4% (12)
None through ICT (e.g., phone, internet, etc.)			39.1% (201)			46.6% (136)			32.8% (62)			3% (1)
Face-to-face psychotherapy usage	–	–		–	–		–	–		–	–	
Yes			61.9% (318)			100% (292)			0% (0)			78.8% (26)
No			38.1% (196)			0% (0)			100% (292)			21.2% (7)
Telepsychology usage	–	–										
Yes			6.4% (33)			0% (0)			0% (0)			100% (33)
No			93.6% (481)			100% (292)			100% (292)			0% (0)

*Note that, due to missing data, not all percentages amount to 100%.*

### Measures

The tool used for gathering data was an online survey that was built by reviewing existing literature and using existing items from questionnaires when were available. Also, an iterative process was followed by the authors, to discuss contents of items. In addition, before making the survey available to participants, it was tested by four volunteers who suggested changes that were implemented. They could judge both the format and functionality of the online survey and the content of the items. Regarding the content of items, they could assess if they were appropriate for the targeted construct and easily understandable.

The survey was responsive to different devices, but we recommended that potential participants complete it using a computer since it was perceived by the research team and users who tested it in advance to be easier. The only inclusion criteria for participation were being older than 18 years and answering completely the questionnaire. In the data collection process, anonymity and confidentiality were guaranteed, and participants provided their consent to participate by accessing the survey and accepting the conditions (i.e., all responses were anonymous, no personal data were gathered, and participants could stop participating at any time). No incentive was offered to participants. The protocol was previously approved by the university’s ethics committee.

The survey assessed the dimensions that are presented below. Questions had to be completed to progress in the survey and move to the next screen (if a question was not answered, the system provided an error message). The user’s IP was not registered to guarantee anonymity; however, the Qualtrics system maintains an opened survey and saves a participant’s progress for a week. So, during this period, if participants stopped and restarted the survey, they were directed to the exact place they were when they left the survey (if they used the same computer and browser).

#### Sociodemographic Variables

Gender was measured with a categorical question with two response options: (1) man and (2) woman. Age was gathered as a continuous variable. Education was gathered by using a categorical question with the following options: (1) no studies; (2) primary studies; (3) secondary studies; (4) university studies; and (5) post-graduate studies. Finally, economic status was also gathered with a categorical question with the following options (in euros): (1) lower than 600; (2) 600–999; (3) 1,000–1,499; (4) 1,500–1,999; (5) 2,000–3,000; (6) higher than 3,000.

#### eHealth Skills and Experience of eHealth

eHealth skills were measured through the 7-item scale: eHeals (Pérez et al., 2015). It was a Likert scale with five response options (1 strongly disagree–5 strongly agree). An example of an item was: “I know what health resources are available on the internet.” The Cronbach’s alpha was 0.91.

eHealth experience was a self-developed scale. It was composed of a general statement: “From the following options, check the ones you have used for some situation of psychological distress (check as many as you consider applicable),” with the following options: “I have visited web pages; I have communicated with professionals by email or chat; I have communicated with professionals through videoconferences; I have used some mobile application (Apps); I have contacted professionals by telephone (telephone therapy); None through ICT (e.g., phone, internet, etc.); and others.” The final value was the sum of all the selected options.

#### Face-to-Face and Telepsychology Use

It was assessed by using the two questions: Have you ever attended any kind of face-to-face psychological therapy? Have you ever attended any kind of online psychological therapy? Two answer options were provided: (1) yes and (2) no.

#### Telepsychology Efficacy, Advantages, and Barriers

Telepsychology efficacy was also measured using a self-developed, 5-point Likert scale. It included the general statement: “Please indicate to what extent you think telepsychology can be effective for the following issues,” with eight items reflecting the most common presenting problems in psychotherapy. More specifically, the items were: (1) improvement of mood disorders (e.g., depression and anxiety), (2) improvement of relational problems (e.g., couple or family problems), (3) improvement of work-related stress problems, (4) health problems (e.g., chronic pain, diet, and fibromyalgia), (5) personal growth issues, (6) mild psychological problems (interfering little with daily life), (7) moderate psychological problems (interfering moderately with daily life), and (8) severe psychological problems (interfering seriously with daily life). The response options ranged from 1 (not at all) to 5 (very much so).

Telepsychology advantages were assessed by the questions: “Please indicate the different advantages that might motivate you to use telepsychology.” According to the literature (e.g., Briffault et al., 2008), the possible answer options were: (1) lower economic cost, (2) the possibility of receiving treatment from home, (3) access to specialized treatment, (4) greater anonymity, (5) as a complement to face-to-face psychotherapy, and (6) none of the above. All “yes” responses were given a value of 1, except for the last option (none of the above), which was given a value of 0. The sum of the marked (“yes”) options was the index that represented perceived telepsychology advantages.

Similarly, for barriers we use the question “Please indicate to what extent the following elements would present a barrier to doing online psychotherapy,” with nine items that reflected the main barriers identified in the literature (e.g., Olfson et al., 2002; Briffault et al., 2008). These items were: (1) it would prevent me from having close or warm contact with my therapist, (2) it would prevent me from expressing my emotions or feelings, (3) I would not be able to pick up on the therapist’s non-verbal language well, (4) the therapist would not understand my non-verbal language well, (5) there would be online confidentiality risks, (6) I would not have enough connection speed or the connection would cut out, (7) there is scarce scientific evidence for the efficacy of telepsychology, (8) there is scarce legal regulation, and (9) I lack the knowledge or resources required to videoconference. The response options varied from 1 (not at all) to 5 (very much so). The mean of the items was the index that represented perceived telepsychology barriers.

### Data Analytic Strategy

All statistical analyses were performed using SPSS statistics software, version 22.0 (IBM Corp.). All tests were two-sided with a type I error set at 0.05. Cluster analysis was performed to examine the patterns on the basis of the experience of using telepsychology and face-to-face psychotherapy. Clustering involves sorting cases or variables according to their similarity in one or more dimensions and producing groups that maximize within-group similarity and minimize between-group similarity (Henry et al., 2005). So, participants’ data were clustered based on their personal experience in face-to-face psychotherapy and telepsychology by applying a two-step cluster analysis procedure. Loglikelihood measured the distance between face-to-face psychotherapy and telepsychology. The clustering criterion was Schwarz’s Bayesian Criterion (BIC). The cluster analysis was a preliminary step to discriminant analysis. It allowed to identify the different groups that were examined in the following discriminant analysis.

Later, the baseline characteristics of all participants are presented in terms of their perception of telepsychology barriers, advantages and efficacy as frequencies and associated percentages for categorical parameters, and as a mean (SD) for continuous variables. These same analyses were performed by cluster. In addition, several ANOVAs were conducted to examine if these differences were significant among clusters, followed by a Tukey *post-hoc* test to determine the specific differences among clusters. Finally, we performed a discriminant analysis. This is a method used in a multi-group setting to demonstrate whether several independent variables (nominal and/or continuous) are related to group membership and how they are combined to better understand group differences (Raykov and Marcoulides, 2012). In fact, the use of cluster analysis in combination with discriminant analysis provides further validation of clusters (McIntyre and Blashfield, 1980). More specifically, we compute discriminant analysis to test the unique differentiating role of sex, age, education, economic status, eHealth skills and eHealth experience across the patterns or groups identified previously in the cluster analysis.

## Results

### Clusters Analyses: Face-to-Face Psychotherapy and Telepsychology

Before performing cluster analysis, an analysis was carried out to assess missing data. Missing data are a common problem in psychological research because participants do not always answer all the survey questions. An inappropriate treatment of missing data can generate biased statistical inferences when statistical techniques are used (Fichman and Cummings, 2003). Hence, an appropriate treatment of missing data is necessary. A crucial decision is whether missing data represent more than 5%. In our study, the percentage of missing data was small. Most variables presented a percentage of around 0%, except for the case of economic status, which showed a value of 4.7%, a figure that is lower than the cutoff criteria. Thus, we could conclude that the missing data had no significant effect on the results of our study (Fichman and Cummings, 2003).

The results suggested three clusters in terms of experience of face-to-face psychotherapy and telepsychology, using a two-step procedure, as suggested by Hair and Black (Hair et al., 2000). The clustering criterion was Schwarz’s Bayesian Criterion (BIC). The two-step procedure (combining hierarchical and non-hierarchical methods) efficiently formed three clusters. We named these clusters, considering percentages of experience in face-to-face psychotherapy and telepsychology. Cluster 1 was called face-to-face psychotherapy and comprised 57% of the sample (*n* = 292), showing experience in face-to-face psychotherapy but none in telepsychology. Cluster 2 was called non-therapy and comprised 36.8% of the sample (*n* = 189), showing no experience in therapy, whether face-to-face or telepsychology. Cluster 3 was called combined therapy and comprised 6.4% of the sample (*n* = 33), in this case showing experience in face-to-face psychotherapy and telepsychology. In order to examine the relationship between experience in face-to-face psychotherapy and telepsychology (two categorical variables) and clusters (two-way contingency table analyses), the chi-square for independence was computed. The results showed significant differences between clusters regarding experience in face-to-face psychotherapy [Pearson’s c2 (d.f. = 2, *n* = 514) = 490.62, *p* = 0.00] and experience in telepsychology [Pearson’s c2 (d.f. = 2, *n* = 514) = 514.00, *p* = 0.00].

### Perception About Telepsychology: Telepsychology Efficacy, Advantages, and Barriers

Table 2 shows means, standard deviations and frequencies of variables related to people’s subjective perception of telepsychology. Overall, people perceived that telepsychology has a few advantages, a moderate level of barriers and is moderately efficient. However, ANOVA results showed significant differences in telepsychology advantages [*F*(2, 470) = 2.49, *p* = 0.03], barriers [*F*(2, 470) = 3.52, *p* = 0.03], and efficacy [*F*(2, 478) = 5.24, *p* = 0.00], depending on the usage patterns identified above. More specifically, Tukey *post*-*hoc* tests only showed significant differences between face-to-face psychotherapy and non-therapy patterns, with people from non-therapy patterns perceiving higher advantages to telepsychology (differences value = –0.31; *p* = 0.03). The combined therapy pattern perceived a lower number of barriers compared to the other two patterns (face-to-face psychotherapy pattern, differences value = –0.46; *p* = 0.02, non-therapy pattern, differences value = –0.43; *p* = 0.04). Finally, the combined pattern perceived higher efficacy than people from the face-to-face psychotherapy pattern (differences value = –0.58; *p* = 0.01).

**TABLE 2:** Descriptive and frequency analysis of perception of telepsychology: advantages, barriers, and efficacy.

	Total			Cluster 1: face-to-face psychotherapy			Cluster 2: non-therapy			Cluster 3: combined therapy		
	Mean	SD	Freq. (N)	Mean	SD	Freq. (N)	Mean	SD	Freq. (N)	Mean	SD	Freq. (N)
**Telepsychology Advantages**	2.61	1.28	–	2.48	1.22	–	2.79	1.38	–	2.82	1.09	–
Lower economic cost	–	–	59.7% (307)	–	–	59.6% (174)	–	–	60.3% (114)	–	–	57.6% (19)
Possibility of doing it from home	–	–	65% (334)	–	–	62.7% (183)	–	–	68.3% (129)	–	–	66.7% (22)
Access to specialized treatment not available in my geographical location	–	–	49.2% (253)	–	–	46.6% (136)	–	–	50.8% (96)	–	–	63.6% (21)
Greater discretion/anonymity	–	–	27.2% (140)	–	–	21.9% (64)	–	–	37% (70)	–	–	18.2% (6)
As a complement to face-to-face psychotherapy	–	–	38.9% (200)	–	–	42.8% (125)	–	–	33.3% (63)	–	–	36.4% (12)
None	–	–	6.6% (34)	–	–	7.5% (22)	–	–	6.3% (12)	–	–	15.2% (5)
**Telepsychology Barriers*^a^***	3.04	0.90	–	3.08	0.89	–	3.05	0.88	–	2.61	1.00	–
It prevents having close or warm contact with the therapist	3.56	1.40	68.5% (352)	3.67	1.41	62% (181)	3.50	1.33	67.7% (128)	2.79	1.42	45.5% (15)
The therapist does not capture my non-verbal language well	3.08	1.45	58.4 (300)	3.15	1.46	71.6% (209)	3.08	1.41	56.6% (107)	2.32	1.33	36.4% (12)
It prevents me from expressing emotions or feelings	3.60	1.29	72.2% (371)	3.68	1.29	76.4% (223)	3.55	1.27	68.8% (130)	3.07	1.36	54.5% (18)
It does not capture the therapist’s non-verbal language well	3.77	1.23	75.9% (390)	3.86	1.20	79.8% (233)	3.73	1.24	72.5% (137)	3.18	1.36	60.6% (20)
Confidentiality risks when using the internet	2.59	1.48	42.8% (220)	2.50	1.45	41.8% (122)	2.73	1.52	45.5% (86)	2.68	1.59	36.4% (12)
Insufficient connection speed/connection cuts	3.06	1.42	57.4% (295)	3.09	1.43	61.3 (179)	2.98	1.42	50.8% (96)	3.18	1.25	60.6% (20)
Little scientific evidence of the effectiveness of online therapies	2.65	1.28	51.2 (263)	2.66	1.31	53.1 (155)	2.73	1.22	53.4% (101)	2.11	1.26	21.2% (7)
Little legal regulation	2.83	1.34	54.9% (282)	2.79	1.38	54.5% (159)	2.99	1.25	60.3% (114)	2.29	1.27	27.3% (9)
Lack of knowledge or means to hold a videoconference	2.25	1.36	37% (190)	2.28	1.37	38.7% (113)	2.28	1.34	37.6% (71)	1.89	1.37	18.2% (6)
**Telepsychology Efficacy^a^**	3.09	1.02	–	2.99	0.99	–	3.18	1.04	–	3.57	1.09	–
Improve mood problems (depression, anxiety, etc.)	3.00	1.29	62.1% (319)	2.81	1.24	57.9% (169)	3.21	1.30	67.2% (127)	3.64	1.31	69.7% (23)
Improve relationship problems with others (partner, family, etc.)	3.06	1.21	64% (329)	2.96	1.16	62.7% (183)	3.16	1.25	65.1% (123)	3.54	1.26	69.7% (23)
Improve work stress problems	3.35	1.26	71.4% (367)	3.21	1.23	69.9% (204)	3.49	1.29	74.1% (140)	3.86	1.18	69.7% (23)
Health problems (chronic pain, fibromyalgia, diet, etc.)	2.78	1.33	53.1% (273)	2.74	1.27	53.8% (157)	2.77	1.40	50.8% (96)	3.32	1.33	60.6% (20)
Personal growth	3.47	1.25	73.7% (379)	3.40	1.22	74.7% (218)	3.50	1.27	73% (138)	3.96	1.35	69.7% (23)
Mild problems (interferes little in daily life)	3.73	1.27	76.5% (393)	3.70	1.25	78.4% (229)	3.72	1.30	74% (140)	3.96	1.23	72.7% (24)
Moderate problems (interfering moderately with daily life)	3.04	1.23	63.4% (326)	2.93	1.20	62.7% (183)	3.14	1.25	63% (119)	3.54	1.26	72.7% (24)
Severe problems (interfering intensely in daily life)	2.27	1.34	38.5% (198)	2.13	1.27	36% (105)	2.43	1.41	41.8% (79)	2.75	1.32	42.4% (14)

*^a^The frequencies refer to percentage of participants with a rate equal to or higher than 3.*

The three main advantages of telepsychology were: (a) lower financial cost, (b) possibility of doing it from home, and (c) access to specialized treatment not available in my geographical location. They were mentioned by more than 50% of participants. However, we can find differences among usage patterns. The possibility of doing psychotherapy from home was the most highlighted advantage in the three usage patterns, followed by its lower financial cost in the face-to-face psychotherapy and non-therapy patterns, and placing the combined pattern in third position. However, in the latter, the second most frequently mentioned advantage was access to specialized treatment not available in my geographical location, which was in the third place for the other two usage patterns.

All participants, irrespective of their usage pattern, agreed with the main telepsychology barriers: “It prevents me from expressing emotions or feelings” and “I do not capture the therapist’s non-verbal language well,” which were mentioned by more than 60% of participants. All usage patterns also mentioned: “The therapist does not capture my non-verbal language well,” but it was more frequent in the face-to-face psychotherapy pattern; “It prevents having close or warm contact with the therapist” was more frequent in the non-therapy pattern; and “Insufficient connection speed/connection cuts” was more frequent among people from the combined pattern.

Finally, around 70% of participants, irrespective of their usage pattern, agreed with telepsychology being perceived as efficacious for “Personal growth,” “Improve work stress problems” and “Mild problems (interfering little in daily life).” In addition, the combined pattern also included “Moderate problems (moderately interfering with daily life)” as a proper target for online psychotherapy. In sum, most participants perceived telepsychology as efficacious, but only for moderate and mild problems.

### Individual Characteristics for Telepsychology Usage: A Discriminant Analysis

Table 3 presents the descriptive analysis (mean and standard deviations) and correlations among sociodemographic and eHealth variables, as well as psychotherapy and telepsychology usage. The results showed that most variables were significantly related, leading to especially high values for the relationships between economic status and age, and eHealth experience and telepsychology usage.

**TABLE 3:** Correlation analysis.

	1	2	3	4	5	6	7	8
Sex	–							
Age	–0.03	–						
Education	0.06	0.03	–					
Economic status	−0.13**	0.41**	0.21**	–				
eHealth experience	–0.02	–0.08	0.03	–0.07	–			
eHealth skills	0.07	0.04	0.18**	0.04	0.15**	–		
Face-to-face psychotherapy usage	–0.02	−0.13**	0.01	0.04	–0.03	–0.01	–	
Telepsychology usage	–0.03	0.01	–0.07	–0.02	−0.41**	−0.11*	0.09*	–

*p = 0.05; **p < 0.01 (two-tailed). Sex (1 man; 2 woman); face-to-face psychotherapy usage (1 Yes; 2 No); telepsychology usage (1 Yes; 2 No).

Table 4 presents extracted canonical discriminant functions and their statistical significance. The discriminant analysis generated two functions that significantly explained variability between groups (clusters). In other words, although Wilks’ lambda presented high values close to 1, chi-squared values were significant; thus, both functions 1 and 2 were significant and show discriminant capacity. If we observe the mean scores of the groups for each discriminant function (centroids), they indicated that the first function maximally differentiates cluster 1 (face-to-face psychotherapy) from cluster 3 (combined face-to-face psychotherapy and telepsychology) and the second one maximally separates cluster 1 (face-to-face psychotherapy) from cluster 2 (non-therapy).

**TABLE 4:** Results of discriminant analysis.

	Mean and standard deviation						Standardized discriminant function Coefficients*^a^*	
	Cluster 1		Cluster 2		Cluster 3		Function 1	Function 2
Sex	1.80	0.39	1.78	0.41	1.81	0.39	0.04	0.15 (0.16)
Age	37.53	9.46	34.99	10.58	36.29	9.40	−0.05	**0.98 (0.73)**
Education	3.90	0.79	4.01	0.83	4.11	0.89	0.05	**−0.26 (−0.32)**
Economic status	2.96	1.34	3.11	1.40	3.18	1.52	0.18	−0.57 (−0.28)
eHealth experience	0.82	0.84	0.91	0.82	2.37	1.07	**0.98 (0.98)**	0.13
eHealth skills	3.28	1.01	3.30	0.97	3.69	1.16	0.09 (0.22)	0.05
% variance							88.1%	11.9%
Canonical correlation							0.40	0.16
Wilks’ lambda							0.81	0.97
Chi-squared (gl)							88.32 (12)**	11.28 (5)*
Centroids of:								
Cluster 1							−0.161	0.120
Cluster 2							−0.023	–0.221
Cluster 3							1.698	0.093

*p < 0.05; **p < 0.01. ^a^In parentheses are the coefficients of a stepwise solution that included only variables entered at the 0.05 significance level (coefficients higher than 0.30 are in boldface).

Regarding the contribution of each discriminant variable to functions, saturations reflected the correlation of each variable and function scores. So, the variables with higher saturations (positive or negative) present a stronger statistical relationship with the function and they can explain the nature of the function. Our results exposed that the first function explained 88.1% of the variance, highly loaded by eHealth experience and moderately but significantly by eHealth skills. So, people who have attended face-to-face psychotherapy (cluster 1) present a lower mean in experience using online health resources (eHealth experience) and in eHealth skills compared to people who have attended combined therapy, understood in terms of face-to-face and telepsychology (cluster 3). The second function explained the 11.9% of variance, highly loaded by age and education, and moderately but significantly by sex and economic status. So, people who have attended face-to-face psychotherapy (cluster 1) seem to be older women and to have a lower level of education and economic status compared to those who do not attend any psychotherapy (cluster 2).

Together, the results of the discriminant analyses corroborate that eHealth experience, eHealth skills, sex, age, education, and economic status help to differentiate among the three patterns of relations between face-to-face psychotherapy and telepsychology. eHealth experience was better at differentiating people who will attend face-to-face psychotherapy or face-to-face combined with telepsychology; whereas sex, age, economic status, and education were better at differentiating people who only attend face-to-face psychotherapy from those who do not attend any type of psychotherapy.

## Discussion

Information and communication technologies (ICT) have increased in importance in the field of psychotherapy, with telepsychology playing a special role in routine practice. However, the social perception and usage of telepsychology, and profile of population who use it has been overlooked. This study aimed to contribute to this understanding by establishing different objectives. The first was to identify usage patterns in terms of whether a person uses telepsychology or not and/or face-to-face psychotherapy. Second, we examined people’s perceptions about the advantages, barriers and efficacy of telepsychology according to identified patterns. Third, some personal characteristics for discriminating between each of the three patterns were identified. These characteristics were sex, age, education, economic status, eHealth skills and eHealth experience.

We accomplished our first research objective, which was to identify the usage patterns of psychotherapy regarding the way in which it is delivered (videoconference or face-to-face). The most populated cluster was the face-to-face psychotherapy only pattern, followed by the non-therapy pattern, and finally the combined therapy, which involved face-to-face psychotherapy and telepsychology. These results showed that Spanish participants do not use telepsychology in an isolated way but rather combined with face-to-face psychotherapy. Thus, there is still a long way to go before the use of ICT takes hold in the psychology field independently of the face-to-face mode.

This study also evidenced that the perception of telepsychology varied among people depending on their usage pattern. People from combined psychotherapy perceived telepsychology as more efficient and with fewer barriers compared to others, especially to people from face-to-face psychotherapy only. Curiously, regarding telepsychology advantages, no differences were found between the combined psychotherapy pattern and the others, but they were observed among people who attended conventional face-to-face therapy and those who did not. It seems plausible to conclude that the perception of barriers and efficacy seems to be critical for people who attend telepsychology in addition to conventional face-to-face psychotherapy. Furthermore, people from the three usage patterns agreed with the main advantages of telepsychology: (a) lower financial cost, (b) possibility of doing it from home, and (c) access to specialized treatment not available in my geographical location. Similarly, they agreed with the telepsychology barriers, referring to its limitation in capturing the therapist’s non-verbal language and to the patients’ expression of their emotions and feelings. Surprisingly, confidentiality concerns were not identified as a relevant barrier. So, it seems that the main barrier to people using telepsychology refers to emotion expression and non-verbal communication, which can have a significant impact on the construction of the therapeutic alliance. Thus, therapists willing to use online videoconferencing should employ additional strategies to overcome perceived barriers and assure the emotional aspects of the therapeutic alliance. Although there is already research and advances related to these issues, for example, emotion recognition systems (e.g., Busso et al., 2004; Mohammadi et al., 2017), these barriers warrant further research on ICT for psychotherapy. Regarding its efficacy, it is worth noting that the majority of the participants perceived online psychotherapy as an efficacious modality, at least for mild to moderate problems.

Finally, our results also showed that some personal characteristics can help to differentiate people in each of these patterns. More specifically, our study evidenced that sociodemographic variables are critical to determine whether a person is going to attend psychological therapy or not, whereas ICT competences and eHealth experience are the critical factors that determine whether a person will attend psychological therapy through videoconference in addition to the conventional face-to-face psychotherapy. People who have higher eHealth skills and eHealth experience are more likely to use face-to-face therapy in combination with telepsychology to a greater extent compared to people with lower eHealth skills and experience, who seem to prefer a face-to-face psychotherapy format. Likewise, older women with a lower level of education and financial resources tend to use more conventional psychotherapy compared to younger men with higher education and financial resources, who tend to attend psychotherapy less often. These results corroborate the relevance of personal characteristics in relation to a preference to use a particular type of psychotherapy or not. These results were congruent with the previous literature on the association of patients’ profiles and use of therapy (e.g., Briffault et al., 2008; Bernecker et al., 2017; Varker et al., 2019).

### Limitations

Despite the contribution of this study to the social perception of telepsychology, it also presents some significant limitations. First, a cross-sectional design was used, therefore a non-causal relationship can be suggested. Longitudinal research is needed to deal with this issue in future research. Second, the sample of this study presented several limitations. On one hand, the sample size is limited, and specially the size of the group combining face to face therapy and telepsychology. Thus, we must take in caution with the extrapolation of results to other samples. On another hand, our data collection method was based on convenience sampling. It was collected through an advisement in our university and researchers’ contacts. Thus, this sample was probably familiar with ICT, and to have higher levels of ICT skills. This method may limit the extrapolation of results to a wider population, because as Van Dijk (2005) suggested, lack of ICT skills would make individuals perceive a difficulty in ICT use, whereas their higher levels would positively affect users in their decision to adopt ICT. However, our sample’s eHealth skills, understood as ICT skills related to health, had a mean level of 3.30 with a standard deviation of 1. It is possible to suggest that there was diversity in the sample in terms of the level of eHealth skills. So, as in other works (e.g., Bakker et al., 2019), it is unlikely that this jeopardizes the validity of our results. It seems more probable that the variables studied and their relationships are similar in other samples. Nevertheless, as we mentioned, given that this study presents a convenience sample, we call for more research on this topic to generalize our results.

Finally, 79.8% of the sample was constituted by women. This composition could have influenced the effect of sex on different telepsychology usage patterns. Further research is needed to replicate and validate our results. Despite its limitations, this paper offers a naturalistic view of telepsychology as perceived by society. This paves the way for future research on patients’ profiles and therapeutic strategies to improve the effectiveness of online psychotherapy, a modality that has significantly increased during recent years (especially after the COVID-19 pandemic) and may establish itself incrementally in the coming years as ICT continue to advance.

### Implications

The main aim of psychotherapy, irrespective of the medium used, is to improve people’s mental health and wellbeing. In this regard, our taxonomical approach provides relevant empirical evidence, facilitating the achievement of this endeavor. In this respect, the inclusion of ICT in health in general, and in psychotherapy in particular, has many significant associated outcomes. Of particular note is its capacity to facilitate access to psychotherapy for people who cannot access face-to-face specialized treatment. Although research seems to suggest that health professionals accept the inclusion of ICT in their services and therefore offer telepsychology, people, in contrast, do not use this service much. It is possible that some people are not attending psychotherapy due to access problems even though they could be solved through ICT. Future research is necessary to gain a better understanding of what factors limit the acceptance of telepsychology in society and, thus, access to telepsychology. Only in this way will health professionals be able to adopt the necessary measures to spread psychological practice to people that need it regardless of the mode of delivery (face-to-face or telepsychology).

Furthermore, our study provides valuable information on some factors that are critical to promoting the use of face-to-face psychotherapy and telepsychology. Whereas, sociodemographic variables (e.g., sex, age, education, and economic status) are critical to determine psychotherapy usage, ICT competences and eHealth experience are critical to determine telepsychology usage. In this vein, both face-to-face psychotherapy and telepsychology should be designed with an emphasis on the patients’ characteristics in order to meet potential patients’ needs and requirements. As Lake and Ondersma (2008, p. 56) pointed out, “identifying the treatments that are most effective for specific clients (i.e., client–treatment matching) is a major goal of research in psychological therapy.”

Finally, this study evidences that the perception of telepsychology in terms of advantages, barriers and efficacy varied among the different patterns. Here, future research is needed to clarify the directionality of this relationship or even its bidirectionality. In other words, is it possible that people change their perception of telepsychology after using it or, conversely, do only people with a positive perception use telepsychology? This question opens up new research lines that could underpin the basis of future practical measures to promote telepsychology in society, focused on improving its social perception or first-time use, for example.

## Conclusion

This study confirmed the existence of three patterns of psychotherapy use based on different media. According to these usage patterns, although there are differences in the perception of telepsychology, there is a general agreement about the main and more frequent advantages, barriers and perceived efficacy. In addition, we also identified some personal characteristics that significantly discriminate among those patterns. Our preliminary typology and the associated characteristics offer a guide for developing more tailored and attractive psychological treatments adapted to the preferences and needs of each type. By identifying distinct patterns of use that may be linked to relevant characteristics, our typology can form a framework which researchers could use to design future studies focused on the next generation of burgeoning telepsychology.

## Data Availability Statement

The data that support the findings of this study are available on request from the corresponding author. The data are not publicly available due to privacy or ethical restrictions. Requests to access the datasets should be directed to BS, beatriz.sora@urv.cat.

## Ethics Statement

The studies involving human participants were reviewed and approved by the Open University of Catalonia. The patients/participants provided their written informed consent to participate in this study.

## Author Contributions

BS, RN, and AM: conceptualization, methodology, and investigation. BS: formal analysis. BS, RN, AM, and MA: writing—original draft preparation, review and editing. All authors have read and agreed to the published version of the manuscript.

## Conflict of Interest

The authors declare that the research was conducted in the absence of any commercial or financial relationships that could be construed as a potential conflict of interest.

## Publisher’s Note

All claims expressed in this article are solely those of the authors and do not necessarily represent those of their affiliated organizations, or those of the publisher, the editors and the reviewers. Any product that may be evaluated in this article, or claim that may be made by its manufacturer, is not guaranteed or endorsed by the publisher.
